# Clinical Biomarkers of Acute Vaso-Occlusive Sickle Cell Crisis

**DOI:** 10.7759/cureus.56389

**Published:** 2024-03-18

**Authors:** Kashish Khurana, Satish Mahajan, Sourya Acharya, Sunil Kumar, Saket Toshniwal

**Affiliations:** 1 Department of Medicine, Jawaharlal Nehru Medical College, Datta Meghe Institute of Higher Education and Research, Wardha, IND

**Keywords:** hemolysis, hypercoagulability, sickle cell crisis, clinical biomarkers, sickle cell disease

## Abstract

It is known that an inherited blood condition called sickle cell disease (SCD) is a result of one gene. A number of blood and urine biomarkers have been determined in association with lab and clinical history for SCD patients. SCD has numerous interacting pathways associated with it, which have been identified by biomarkers. These mechanisms consist of some examples, such as endothelial vasodilation response, hypercoagulability, hemolysis, inflammation, oxidative stress, vascular dysfunction, and reperfusion injury among others. To effectively manage SCD, a comprehensive panel of validated blood and urine biomarkers must be established. Despite its monogenic inheritance, the complex nature of the SCD phenotype has impeded progress in its treatment. However, significant strides have been made in clinical biotechnology, paving the way for potential breakthroughs. In SCD, a panel of verified blood and urine biomarkers must be established, however. Despite monogenic inheritance, the great complexity of the SCD phenotype has hindered progress in its management. With few exceptions, clinical biomarkers of illness severity have been found through epidemiological investigations; nevertheless, systematic integration of these biomarkers into clinical treatment algorithms has not occurred. Furthermore, sickle cell crisis, the primary acute consequence of SCD, has been difficult to diagnose with the biomarkers now in use. Inadequate care and a lack of appropriate outcome measures for clinical research are the consequences of these diagnostic constraints. A new chapter in SCD customized treatment has begun with recent advancements in molecular and imaging diagnostics. Strategies in precision medicine are especially relevant now that molecular therapies are within reach. The significance of biochemical indicators linked to clinical manifestation and sub-phenotype identification in SCD is reviewed in this research.

## Introduction and background

The most common hemoglobinopathy in the world, sickle cell anemia (SCA), is the most severe variant of a group of hereditary illnesses involving the β-globin gene. The homozygous form of a single mutation (adenine > thymine) on the 17^th^ nucleotide from region 15.5 of chromosome 11 is the cause. As a result, valine is produced instead of glutamic acid, and hemoglobin S (HbS), a tetrameric protein, is formed [1]. The changes brought about in red blood cells (RBCs) due to HbS polymerization under low oxygen are substantial. Such changes affect how these cells interact with platelets, leukocytes, and cells that line the blood vessels. This altered state can lead to low blood flow, injury from the return of normal blood flow, and blockage in small blood vessels. Each of these factors contributes to longer clotting processes [1,2].

This single gene change significantly affects the body's defense response to injuries caused by restricted and returned blood flow and delayed red cell breakdown. These impacts, in turn, shape how SCA appears in patients. In addition, SCA presents a complicated pattern of slowly worsening harm to various organs, even at rest. This is interrupted by sudden painful events due to blockages in the blood vessels. These painful events are known to worsen the SCA state, which is inclined towards producing inflammation [1,2,3].

In individuals with SCD, one of the most debilitating and severe complications is vaso-occlusive crisis (VOC). Sickle erythrocytes attach themselves on the endothelium to the leukocytes that have become immobile, leading to microvascular occlusion and tissue ischemia, which can cause VOC. Chronic debilitating arthritis resulting from osteonecrosis affecting the joints, progressive retinopathy, chronic renal failure, an elevated risk of stroke, and a shorter lifespan are all caused by recurrent vaso-occlusion [3,4,5]. Effective management of SCD and a lower death rate are both possible with early identification. As a result, several methods have been created to accurately and completely identify sickle cell disease and its carrier states. These methods include screening tests like sickling tests, peripheral blood smears, and total blood counts; confirmatory tests like hemoglobin separation methods; and genetic studies, which are more costly and require specialized workers to perform in centralized labs [6.7]. Those with SCD need better solutions and biomarkers to make things easier for them. Discovering useful treatments can greatly enhance the lifestyle of those living with SCA.

## Review

Sickle­ cell disease (SCD) happens when there's not enough oxygen. It turns the RBCs sickle-shaped, making them stick together more. This creates blockages in our major vessels. SCD is inherited in families and happens due to HbS. HbS is an abnormal kind of hemoglobin that makes cells stiff [2,4]. VOCs can be extremely painful, causing high fevers and damaging vital organs by disrupting blood flow and oxygen supply to tissue. Such episodes may occur frequently and subtly, ultimately leading to repeated hospitalizations and a diminished quality of life [3,4].

Prevalence and global distribution: SCD is a worldwide health concern. It's typically found in areas once infected by malaria. These include places like sub-Saharan Africa, the Middle East, India, and the Mediterranean. This is because people with sickle cell trait may resist severe malaria better [5,6]. The World Health Organization (WHO) says each year around 300,000 newborns have severe hemoglobin defects. SCD affects lots of African Americans. There could be as many as 100,000 case­s in the United States [5].

SCA and hemoglobin variants: SCA happens when people get two copies of the HbS gene. It's the most common, and the worst kind of SCD (HbSS). There are other types that come from blending HbS with other abnormal hemoglobin genes. These are called HbSC and HbSβ-thalassemia [7].

Clinical manifestations and complications: SCD is known for its characteristic crisis, vaso-occlusive crisis (VOC). This crisis happens when misshaped RBCs jam up in blood vessels. This can result in severe pain, organ damage, and various outcomes. Also, acute chest syndrome, stroke, anemia, and other organ issues may show up in patients with SCD [8,9].

The most frequent and serious consequence of SCD is VOC. Severe pain episodes, fever, and organ malfunction are its hallmarks. This condition is brought on by sickle-shaped RBCs getting stuck in blood vessels. These sickly-shaped red cells, unlike normal round ones, are stiff and clump together. They can stick to the walls of blood vessels and block them. This causes pain, swelling, and organ damage due to reduced or abnormal blood flow to various organs and tissues [4,10]. For patients with SCD, VOC is a big cause of morbidity and mortality. It could lead to severe pain, often in the bones, joints, chest, or abdomen. The pain can be moderate to severe in intensity, requiring hospitalization and analgesics [8,11]. It can also cause tissue damage due to reduced blood flow during VOC, which may cause issues with different tissues and organs like the kidneys, the lungs, the heart, or the brain. There is an increased risk of infection in these patients because of their weakened immunity induced by VOCs. These make the patient’s condition worse by extending the duration of hospital stay [10,11].

This leads to reduced quality of life due to frequent episodes of pain, hospitalization, and organ damage for SCD patients [8]. SCD in most cases is also accompanied by increased health care costs due to VOCs. The frequent hospitalizations, pain management, and treatment of complications place a significant financial burden on patients and healthcare systems [11].

Pathophysiology of SCD

Millions of individuals around the globe suffer from SCD, which is a class of inherited blood disorders. The alterations found in the β-globin gene result in a change in the production of β-globin, whose major role is that of transporting oxygen in the blood [2,3]. The normal β-globin gene provides instructions for making a slightly sticky hemoglobin chain (β-globin A). Nevertheless, due to changes in the β-globin gene in SCD, it is less sticky. It tends more towards clumping and is known as β-globin S [2].

When RBCs with β-globin S are exposed to low oxygen levels, the hemoglobin chains can stick to each other, causing the RBCs to change shape from a disc-like shape to a sickle-like shape. The RBCs shaped like a sickle are not as bendable as normal ones. These might get trapped in small blood vessels. This can block these vessels, affecting the blood supply to tissues and organs [12]. This blockage of blood flow can lead to a variety of symptoms, including severe pain, fever, fatigue, and organ damage. Yet, how severe the symptoms are can change from one person to another and is variable [12]. SCD has no fixed treatment, but there are ways to ease the symptoms and dodge­ serious issues. These methods include analgesics, hydroxyurea (a drug that can cut down the count of sickle-shaped RBCs), and blood transfusion based on hemoglobin levels.

The rationale for studying clinical biomarkers in VOC

Characteristics that may be reliably tested and assessed as markers of diseased conditions, normal physiological processes, or pharmacologic reactions to a therapeutic intervention are known as biomarkers. Therefore, biomarkers can be helpful at several stages of the disease process, ranging from screening, diagnosis, and tracking the progression of an established disease to detecting risk prior to the development of a disease or a consequence. In the latter scenario, biomarkers can forecast treatment response and outcomes (like death). Beyond their application in clinical settings, biomarkers are also helpful in clinical research since they can be used to determine dosage response, offer proxy results for intervention trials, and shed light on molecular pathways [2].

Studying clinical biomarkers in VOC holds immense promise for improving the management and outcomes of SCD. Biomarkers are measurable indicators of biological processes or pathogenic events that can provide valuable insights into disease progression, risk stratification, and treatment response [2,3].

Early Detection and Risk Stratification

Biomarkers offer the potential for early detection of VOCs, enabling timely interventions and preventing hospitalization. Identifying early biomarkers that signal the onset of VOCs would allow clinicians to initiate treatment promptly, mitigating the severity and duration of VOC episodes and reducing the risk of complications [2,13]. Biomarkers can also aid in risk stratification, allowing clinicians to identify patients at high risk of severe VOCs. This information can guide personalized treatment strategies and resource allocation, ensuring that high-risk patients receive the most appropriate and intensive care [13].

Monitoring Treatment Response

Biomarkers can serve as valuable tools for monitoring treatment response and guiding adjustments to therapy. Tracking changes in biomarker levels allows clinicians to evaluate the success of ongoing treatment plans and make informed choices regarding necessary dosage changes or alternate therapies [14]. Biomarkers provide a crucial understanding of the root causes of VOC, allowing for the development of personalized and effective treatments. By honing in on specific biomarkers involved in VOC formation, researchers can focus their efforts on crafting therapies that specifically address these pathways, potentially reducing the occurrence or intensity of VOC [13,14].

Personalized Medicine

Biomarkers hold tremendous potential in unlocking personalized treatment strategies for SCD, taking into account unique patient characteristics and biomarker profiles. By tailoring treatment plans to each individual, this method can increase treatment effectiveness, reduce side effects, and ultimately elevate patient outcomes [13,14]. Overall, studying clinical biomarkers in VOC holds immense potential for revolutionizing the management of SCD. By providing early detection, risk stratification, treatment monitoring, and insights into disease mechanisms, biomarkers can contribute to improved patient care, reduced healthcare costs, and enhanced quality of life for SCD patients [14].

Established Biomarkers

Several biomarkers have been established as having clinical utility in SCD. Fetal hemoglobin F, a naturally produced type of hemoglobin called HbF, doesn't polymerize in low-oxygen ambient environments. VOCs are linked with increased HbF levels, which prevent HbS aggregation [15]. α-thalassemia is caused by the loss of any one or multiple α-globulin genes genetically. It is almost like HbF, and the resultant reduction of HbS fraction in RBCs also lowers the risk for VOC [16].

A good example of another marker is C-reactive protein (CRP), which is elevated during VOCs. Increased VOCs occur more often in patients whose CRP levels are high and who stay in a hospital for longer periods, making them prone to death [17]. Adhesion molecules are proteins that promote adherence between cells. High concentrations of vascular cell adhesion molecule 1 (VCAM-1) and P-selectin are correlated with an enhanced susceptibility to VOC [18,19]. Thrombomodulin is a soluble protein involved in the regulation of blood clotting. Soluble thrombomodulin levels are elevated in association with VOC, death, and endothelial problems [19].

Emerging Biomarkers

The famous biomarkers for SCD are under review in terms of their utility and new biomarkers for use in SCD management are emerging.

MicroRNAs (miRNAs): Non-coding RNA of a small nature that is known to suppress their expression genes. Some of these miRNAs exhibit different expressions in SCD patients and can be a case study for linking the risk or severity of VOCs in the future [20].

Long non-coding RNAs (lncRNAs): Other than coding, RNAs also include lncRNA involved in diverse functions of cells. Some lncRNAs have been associated with SCD and could indicate future VOC biomarkers [21].

Proteomics: The discipline that looks into the protein makeup of an organism is known as proteomics. Proteomic studies indicate various proteins that may work as biomarkers of SCD and lead to early diagnosis and efficient treatment [22].

Metabolomics: By employing metabolomics, a science that involves small molecular weight substances produced in cell processes, it has been discovered that several altered metabolites are associated with SCD. These metabolic products have been suggested to correlate with the risk or severity of VOC [23].

Hemolytic markers

Hemoglobin and Reticulocyte Count

Hemoglobin: RBCs contain hemoglobin, which helps in the transportation of oxygen from the lungs to other parts of the body. In VOC, the hemoglobin level falls due to increased RBC lysis and reduced hemoglobin synthesis [2].

Reticulocytes: Reticulocytes are young RBCs that have not matured yet. Reticulocyte count is a marker for the RBC turnover rate. The reticulocyte can be elevated in cases when the bone marrow is trying to replace RBCs quickly during VOC. The high reticulocyte count reveals that the person’s bone marrow is producing more RBCs, and it can indicate continuous RBC destruction [2,24].

The interpretation of hemoglobin and reticulocyte count during VOC: Interpretation of hemoglobin and reticulocyte count should be based on a clinical picture of an affected individual and other laboratory data obtained during VOC. However, generally, a low hemoglobin level coupled with a high reticulocyte count indicates continued destruction of RBCs and increased production in bone marrow. This is usually observed in the commencement phases of VOC [2,24]. With progression in VOC, the bone marrow may become exhausted, leading to a reduction in the levels of reticulocytes. Low hemoglobin levels with low reticulocytes suggests very severe anemia and poor RBC production. The presence of this pattern usually indicates a late stage of VOC [25].

Monitoring hemoglobin and reticulocyte count: In VOC, hemoglobin and reticulocyte count are typically evaluated periodically as indicators of response to therapy and severity of crisis. They may even be used in recognizing patients prone to complications like acute chest syndrome/stroke [2]. However, it is worth keeping in mind that VOCs may also affect hemoglobin and reticulocyte count due to infection, dehydration, or blood loss. Hence, such tests should be analyzed based on the whole clinical history. Both hemoglobin and reticulocyte counting provide a reliable way of assessing VOC in SCD. Such examinations are useful for gauging how acute it is, finding out whether patients have any complications, as well as guiding on the type of therapy [2,24].

Haptoglobin Levels

Haptoglobin is a protein that attaches to unbound hemoglobin within the plasma. Haptoglobin guards the body against free hemoglobin and its toxic effects. Haptoglobin levels in the blood may also lower during VOC. VOC induces the lysis of RBCs, releasing free hemoglobin into the blood [25].

Haptoglobin levels and VOC severity: Haptoglobin level studies indicate it as a sign of VOC gravity. Individuals who have little or no haptoglobin suffer more serious VOC attacks. It could be attributed to higher concentrations of free hemoglobin in the blood that result from low haptoglobin levels, leading to tissue damage and inflammatory processes [25,26].

Haptoglobin is a potential biomarker of VOC. Haptoglobin levels may help ascertain the path of VOC development, as well as the future risk of complications. Further studies are required to comprehend completely the influence that haptoglobin has on VOC and how to make better VOC management based on haptoglobin levels [25,26].

*Unconjugated Bilirubin and Serum Lactate Dehydrogenase (LDH) Levels* 

Unconjugated bilirubin and serum LDH levels are two significant markers of RBC lysis. In the phase of VOC, unconjugated bilirubin and LDH may be elevated in the bloodstream. This occurs due to VOC as it triggers hemolysis, leading to an increased concentration of unconjugated bilirubin and LDH in the bloodstream. Hemolysis is indicated by increased unconjugated bilirubin and LDH levels. The two tests are, nevertheless, not the same concerning sensitivity and specificity.

Specificity in the case of hemolysis manifests itself as unconjugated bilirubin. High levels of unconjugated bilirubin are more likely to result from hemolysis compared to other factors. Nevertheless, it has been observed that unconjugated bilirubin is also a less specific indicator of hemolysis. This implies that normal unconjugated bilirubin does not exclude hemolysis [27]. Hemolytic activity is more indicative in LDH markers. Put simply, high levels of LDH can be more attributed to hemolysis than other causes. On the other hand, LDH is a less specific indicator of hemolysis. High levels of LDH may therefore also be due to other reasons including liver damage and skeletal muscle injury [6,28]. Therefore, unconjugated bilirubin and LDH are usually combined for the diagnosis of hemolysis. An elevated level of unconjugated bilirubin in addition to an increased LDH level is an indication of hemolysis [27,28]. Unconjugated bilirubin and LDH are higher in VOC for a reason, that is, in the course of the crisis, these cells break down. An escalating measurement of these markers reflects the degree of hemolysis that has developed.

Inflammatory markers

Pro-inflammatory Cytokines

Interleukin 6 (IL-6) and tumor necrosis factor alpha (TNF-α) are proliferative enzymes that contribute greatly to VOC. VOC is characterized by tissue injury and inflammation. Activated immune cells and endothelial cells release these cytokines upon exposure to VOC. 

IL-6 and TNF-α induce the production of vasoconstrictor-like endothelial-1, which leads to the constriction of blood vessels. This reduction of blood flow causes tissue and organ pain and contributes to tissue injury known as VOC. The adhesion of leukocytes is aided by pro-inflammatory cytokine production. Adherence and migration of circulating leukocyte cells lead to local inflammation and tissue damage [29].

Stimulating the production of reactive oxygen species (ROS): The pro-inflammatory cytokines lead to increased levels of ROS, which are powerful oxidants that can cause damage within cells and their tissues. The effect of oxidative stress linked with VOC is worsened by ROS [29].

The higher levels of pro-inflammatory cytokines contribute to more serious forms of VOC. Higher levels of IL-6 and TNF-α among patients are related to numerous and severe VOC episodes. A number of approaches are being explored to try and ameliorate the effects of VOC by targeting the pro-inflammatory cytokines involved. These strategies include anti-inflammatory drugs, corticosteroids, cytokine-specific inhibitors, and CRP. Some nonsteroidal anti-inflammatory drugs (NSAIDs) like ibuprofen and naproxen may inhibit the synthesis of inflammatory cytokines. Patients with SCD, however, do not necessarily respond well to NSAIDs, which can also bring about undesirable reactions [24].

Prednisone is a corticosteroid that inhibits the production of pro-inflammatory cytokines. Corticosteroids, however, are associated with many adverse effects and must not be administered liberally in SCD patients. Cytokine-specific inhibitors are various monoclonal antibodies, as well as targeted IL-6/TNF-α inhibitors, at different stages of development for use in clinicals. The use of the drugs can be an alternative method for treating high pro-inflammatory cytokine levels in SCD. Therefore, pro-inflammatory cytokines are crucial in VOC pathogenesis and disease severity in SCD. As such, therapeutic approaches aimed at interfering with the production and expression of these cytokines can potentially facilitate effective management of VOC and improved quality of life in SCD patients.

Role of CRP in VOC

C-reactive protein, or CRP, also known as an acute-phase sensitive inflammatory marker, has been shown to play a role in the pathogenesis of VOC in SCD. CRP is increased as part of the systemic reaction to inflammation, and elevated CRP is frequently seen during VOC episodes [29,30].

Activation of endothelial cells by CRP is responsible for enhanced vascular permeability and leukocyte adhesion. It leads to blood vessel occlusion and tissue hypoxia, which are typical of VOC [30]. CRP may also induce the synthesis of other inflammatory mediators that might intensify the inflammatory cascade making tissue damage worse.

Altering coagulation: SCD results in the activation of coagulation through interactions between CRP, an acute phase reactant, and other coagulation factors thereby promoting thrombosis [30]. It is also worth mentioning that CRP is an inflammatory marker that gives information on the pathogenesis and severity of the VOC in SCD. This could help during clinical decision-making and the formulation of personalized management strategies aimed at enhancing VOC management thereby improving health outcomes amongst SCD patients [30].

Endothelial activation markers

Soluble Adhesion Molecules

The inflammatory process is largely mediated by soluble adhesion molecules like intercellular adhesion molecule 1 (ICAM-1) and VCAM-1, which play an important role in the etiology and development of severity of VOC in SCD. These molecules are known as endothelial cell adhesion molecules, which get expressed on the lining of blood vessels, and they interact with leukocytes or white blood cells for the promotion of adhesion and migration to tissues [18,31]. The levels of soluble ICAM-1 and VCAM-1 are elevated during VOC, thus suggesting the enhancement of endothelial cell activation and leucocyte adhesion.

Soluble ICAM-1 and VCAM-1 contribute to VOC by (i) Promoting leukocyte adhesion: The endothelium adhesion molecules bind to the surface receptors on leukocytes that attach them to the endothelium. Adhesion promotes the accumulation of leukocytes around blood vessels, exacerbating the inflammatory process and causing tissue injury [18]. (ii) Enhancing leukocyte transendothelial migration: Another set of molecules that facilitates this process is soluble ICAM-1 and VCAM-1, which promote transendothelial migration of leukocytes and thus their movement crossing through the endothelium into the surrounding tissue. This constitutes an important phase of this inflammatory process that eventually leads to tissue damage. (iii) Mediating vascular dysfunction: Soluble adhesion molecule interactions on leucocytes may trigger endothelial cells causing dysfunction in the blood vessels, leading to poor flow of blood. This condition may serve as an augmentation factor for the obstruction of the blood flow, that is, VOC [18].

Effects of soluble adhesion molecules over severities of VOC: High concentration of soluble forms of ICAM-1 and VCAM-1 is linked to more intense frequency and severity occurrence of VOC attacks. Higher levels of such adhesion molecules tend to lead to worse pain, prolonged VOC episodes, and increased risks of complications [32].

Therapeutic implications of soluble adhesion molecules: Soluble ICAM-1 and VCAM-1 can be used in gauging the seriousness of VOC and the likelihood of complications. The monitoring of these adhesion molecule levels has a role to play in clinical decision-making as well as in determining an effective treatment plan. Moreover, studies on therapeutic interventions against soluble adhesion molecules for VOC management have been conducted [31]. 

Using antibodies that target ICAM1 or VCAM1 could stop the binding of adhesion molecules with leukocytes, decreasing leukocyte adhesion and migration. Also, researchers are studying small molecules that may work as inhibitors for ICAM-1 and VCAM-1 expression.

Therefore, soluble ICAM-1 and VCAM-1 are some important factors that contribute to the genesis and gravity of VOC in SCD. These adhesion molecules act as important biomarkers as well as targets in the area of managing VOC patients effectively, resulting in better results. Additional studies, therefore, will be necessary to completely unravel how soluble adhesion molecules affect VOC and devise robust interventions aimed at these molecules [31,33].

Nitric Oxide (NO) Metabolites and Impaired Endothelial Function

NO is a key component of vascular homeostasis and endothelium function. However, the production of NO is compromised in VOC during SCD, where it contributes to the pathophysiology of the same [32,33]. NO acts as a potent vasodilator that causes relaxation of the smooth muscles in blood vessels that allow for high levels of blood flow to tissues. It also suppresses platelet formation and stickiness of white blood cells, which stops the formation of clots and inflammation as well. NO is secreted for the most part by endothelial cells and this plays an important role in preserving endothelial function [33,34].

During VOC, several factors contribute to impaired NO production in endothelial cells, including oxidative stress, inflammation, and hemolysis [33]. Impaired NO production leads to endothelial dysfunction, characterized by vasoconstriction, increased platelet adhesion and aggregation, enhanced leukocyte adhesion, and inflammation.

Since NO-mediated vasodilation is reduced, this results in vasoconstriction that curtails blood flow and tissue oxygenation. Since NO does not exhibit anti-platelet effects, there are more chances for platelet adhesion and aggregation, leading to the formation of thrombus and vessel occlusions. Without the anti-inflammatory effects of NO, leukocytes stick to and move into the vascular wall, sustaining and worsening inflammation due to tissue injury [33].

Restoring NO bioavailability and improving endothelial function have emerged as promising therapeutic strategies for VOC management. These measures include L-arginine supplementation, antioxidant therapy, and anti-inflammatory medications. L-arginine is a substrate for endothelial nitric oxide synthase (eNOS), which can boost NO synthesis. Oxidative stress can be reduced by antioxidants, which in turn help guard NO against ROS scavenging. These anti-inflammatory agents can prevent or minimize the downregulation of eNOS and inflammation, which could facilitate the generation of more NO [33].

The mechanisms leading to the VOC in SCD include impaired NO production and endothelial dysfunction. A successful approach to treating VOC could entail restoring NO bioavailability and improved endothelial function in an attempt to minimize the condition’s impact on patients. The approaches need further optimization for a complete comprehension of how NO metabolism affects VOC [32,33].

Coagulation markers

D-dimer, a Thrombus Degradation Product and a Marker of Coagulation Activation

D-dimer functions as an indicator of a high level of coagulation activity, and it is highly sensitive. Coagulation process amplification during VOC in SCD develops due to endothelium injury, inflammatory process, and blood flow impairment. This increased activation of coagulation results in fibrin clots, which are broken down into D-dimer [34,35]. High levels of D-dimer in SCD-inflicted patients signal a higher probability of VOC, which can be correlative with the severity of a VOC episode. As they happen, D-dimer levels increase and then decrease as the crisis begins to subside. Elevated D-dimer levels in VOC can have several consequences like increased risk of thrombosis, vascular dysfunction, and organ dysfunction.

D-dimer is a specific marker for fibrinolysis or the process that causes the breakage of fibrin clots. High D-dimer concentrations are indicative of ongoing fibrinolysis, linked with thrombosis, among other phenomena related to the risk of thromboembolism - the formation of undesired blood clots [34]. Release of these fibrin degradation products can lead to pro-inflammation and pro-coagulation effects that worsen vascular dysfunction, thus contributing to VOC severity. The blood sample is collected by venepuncture [35]. Impairment of blood flow and oxygen delivery to some organs, especially lungs, kidneys, and brain, occurs as a result of thrombosis and vascular dysfunction [34].

Therefore, elevated levels of D-dimer can be used in the diagnosis and evaluation of patients with VOC episodes. It is possible that D-dimer levels could be utilized in estimating the chances of developing acute chest syndrome or stroke following VOC. Measured D-dimer may indicate that the therapies reduce clotting activation and improve vascular functioning [34]. D-dimer is a marker of coagulation activation that would be useful in screening VOC in SCD patients. As such, D-dimer level elevation is associated with VOC severe manifestations and complications and has a predictive value as a clinical decision-maker and in terms of treatment optimization.

Thromboxane and Prostacyclin Imbalance

There exist two antagonistic eicosanoids, namely thromboxane A2 (TxA2) and prostacyclin (PGI2), which help in maintaining vascular homeostasis. In particular, a number of researchers have proposed that TxA2 is a potent vasoconstrictor and a platelet aggregator, while PGI2 acts as a vasodilator and a platelet inhibitor. An imbalance of TxA2 versus PGI2 plays a significant role in VOC pathogenesis in SCD patients [36]. During VOC, the balance between TxA2 and PGI2 shifts in favor of TxA2, leading to vasoconstriction, platelet aggregation, and endothelial activation [36,37].

Factors contributing to TxA2-PGI2 imbalance: Several factors that contribute to the TxA2-PGI2 imbalance in SCD and VOC include endothelial activation, inflammatory mediators including TNF-α, and oxidative stress [36].

The TxA2-PGI2 imbalance significantly impacts VOC by exacerbating pain, promoting tissue ischemia, and increasing the risk of complications. The painful effects of VOC are based on the TxA2-mediated vasoconstriction and aggregation of the blood cells (platelets) [36]. Because of vasoconstriction, tissue ischemia occurs, which leads to inadequate oxygen delivery and hence organ dysfunction or death. Thrombosis, acute chest syndrome, and stroke risk are significantly high due to the hypercoagulable state resulting from TxA2-PGI2 imbalance [36].

TxA2-PGI2 imbalance is one of the most important factors contributing to the generation and intensity of VOC in SCD. Such an approach may improve vascular function, decrease the frequency and severity of VOCs, and improve the overall health and quality of life in SCD patients. More studies are required for perfecting this approach and clarification of the mechanism through which TxA2 and PGI2 cause VOC [36].

Thrombotic Events During VOC

Formation of thrombi or blood clots is known as thromboembolic complications and it is a serious risk in VOC for SCD patients. The rise in inflammation, activated endothelium cells, and hypercoagulable conditions are seen during VOC. These events contribute to thrombosis both venous and arterial. Such clots may cut down upon blood circulation to different organs which could lead to potentially life-threatening problems including acute chest syndrome, stroke, and pulmonary thromboembolism [10].

Trends and risk factors of thrombotic events in VOC: Several factors increase the risk of thrombotic events during VOC, including the severity of VOC, history of thrombotic events, female sex, and elevated inflammatory markers [10].

The risks associated with thrombotic events among VOC: Thrombotic events during VOC can have severe consequences, including acute chest syndrome, stroke, and pulmonary embolism. Acute chest syndrome is among the most common and potentially fatal complications of SCD associated with chest pain, shortness of breath, and fever. Acute chest syndrome may also occur as a consequence of a thrombotic event in the pulmonary circulation. A stroke is a deadly neurological problem that happens as a result of an interruption in the flow of blood towards the brain. Stroke is associated with thrombotic events in the cerebral circulation in SCD patients. Pulmonary embolism occurs as a result of a blood clot migrating from another part of the body and leading to the obstruction of an artery of the lung. It can, however, lead to lethal pulmonary embolism in SCD cases [4,10].

Reducing the risk of thrombotic events in VOC: The management of VOC in order to avoid thrombotic events is one of the most important things to do in SCD patients [10,38]. Several preventive measures can be implemented, including early diagnosis and treatment of VOC, hydration, anticoagulation therapy (like hydroxyurea), antiplatelet therapy, and additional preventive measures like regular physical activity, smoking cessation, and controlling weight [38]. A thrombotic event associated with VOC is one of the most serious complications of SCD and threatens life. It is important to understand the risk factors for thrombotic events, implement preventative strategies, as well as provide prompt diagnosis and treatment of VOC episodes to enhance the management of SCD and reduce the risk of these complications [10,38].

Imaging biomarkers

Advanced imaging techniques, such as MRI and Doppler ultrasound, play a vital role in detecting CNS aneurysms. MRI and Doppler ultrasound are advanced diagnostic imaging techniques used for VOC in SCD. Such methods give a lot of evidence about the magnitude and intensity of VOC periods for clinicians to plan treatment strategies and evaluate patient’s progress [39,40].

MRI in VOC

MRI provides an overall picture of each person’s soft tissues inclusive of the bone marrow, blood veins, and organs. During VOC, MRI can be used to detect bone marrow infarcts, assess the extent of tissue ischemia, and identify complications. The bone marrow in infarcts occurs as a result of blockage of major blood vessels resulting in the lack of blood flow. Because of this, it is possible for MRI to detect an infarct during the time when the first stages of VOC occur, thereby ensuring that preventive measures are taken at the best possible time and with appropriate intensity. The areas of tissue not getting enough oxygen as a result of poor circulation can be displayed by MRI in order to determine how serious the case of the VOC is, and thereby help make the right therapeutic decisions [40]. Complications of VOC can be seen on MRI including acute chest syndrome, stroke, and pulmonary embolism [40].

Doppler Ultrasound in VOC

Doppler ultrasound employs sound waves to gauge the velocity as well as the direction of blood flow. During VOC, Doppler ultrasound can be used to assess blood flow in major arteries. In this context, it is evident that Doppler ultrasound also evaluates the flow of blood in the carotid, femoral, and renal arteries for identification of areas of stenosis or occlusion that may have contributed to VOC [39]. In addition, Doppler ultrasound is useful in tracking the effects of any medication regimen like anti-coagulating therapy by determining changes in the flow pattern [40]. Deep vein thrombosis (DVT) is also an important complication of pulmonary embolism and can be detected by the Doppler ultrasound [40,41].

Role of MRI and Doppler Ultrasound

Both MRI and Doppler ultrasound offer vital knowledge on VOC. MRI provides a detailed picture of the soft tissues in the body, while Doppler ultrasound is concentrated on the blood flow mechanism. The combination of the above-mentioned techniques helps in the evaluation of VOC and its co-morbidity. Using advanced imaging methods like MRI and Doppler ultrasound can aid in diagnosing and managing VOC in SCD. These methods give relevant data on the size and intensity of VOC incidents, enabling physicians to form educated treatment plans and boost patients' results [39,40,42].

Vaso-occlusion, the hallmark of VOC, can be assessed through various clinical and laboratory parameters like clinical history, physical examination, laboratory tests, and various imaging studies [40]. A comprehensive clinical approach for assessing organ involvement and vaso-occlusion includes obtaining the history, conducting physical examination, performing laboratory tests, and imaging. Through this multifaceted approach, clinicians can properly assess the magnitude and intensity aspects of VOC, which informs treatment, decision-making, and monitoring responsiveness to therapy [10,43].

The assessment of organ involvement and vaso-occlusion has significant implications for treatment decisions during VOC. Specific organ involvement also points to the need for a particular treatment, such as oxygen therapy in case of lung involvement or diuretics when renal involvement is detected [14,18]. Hospitalization of severe vaso-occlusion and associated organ dysfunction is usually required along with intravenous hydration, analgesia, and intensive supportive care [10,15]. Assessment of organ involvement and vaso-occlusion should be done continuously in order to determine patient response to therapy with a view to adjusting intervention appropriately. Managing VOC in SCD critically includes evaluation of any organ involvement and vaso-occlusion. Through the application of the comprehensive measurement approach that combines different assessment strategies, physicians are able to guide treatment options, enhance the patient's condition, and avoid adverse effects related to the voice onset characteristics [10,18].

Limitations

Despite the significant progress in identifying and utilizing biomarkers for VOC in SCD, several limitations remain. These existing biomarkers like CRP, LDH, and D-dimers do not specifically indicate the incidence of the disease VOC. Other conditions may also lead to increased levels of these markers, leaving the diagnosis of VOC inconclusive [17,44]. Threshold limits for biomarkers may be different in every individual, which makes it hard for standardization of VOC diagnosis [15-17,45,46]. Despite this, biomarkers are important in indicating the presence and severity of VOC but might be unable to accurately predict the occurrence of future episodes and their complications [6].

Inadequate coverage of VOC pathophysiology: Although existing biomarkers mainly represent inflammatory/coagulative aspects of VOC, they do not fully consider these complex biological processes involved in VOC [10,22]. Therefore, ongoing research seeks to develop new biomarkers that are more specific, sensitive, and predictive of VOC. Moreover, attempts are being made to include several biomarkers in combined panels which offer a broader overview of VOC etiopathogenesis and risk stratification [10,11].

Limitations of existing biomarkers used in VOC in SCD have been highlighted in Table 1.

**Table 1:** Summary of limitations of existing biomarkers used in VOC in SCD VOC: Vaso-occlusive crisis; SCD: Sickle cell disease; CRP: C-reactive protein; LDH: Lactate dehydrogenase

Limitation	Description
Lack of specificity	Biomarkers like CRP, LDH, and D-dimer can be elevated due to other conditions, making VOC diagnosis challenging
Variability in marker levels	Biomarker levels vary among individuals, making it difficult to establish standardized thresholds for VOC assessment
Limited predictive value	Biomarkers may not accurately predict the risk of future VOC episodes or complications
Inadequate coverage of VOC pathophysiology	Existing biomarkers focus on inflammation and coagulation but don't fully capture VOC's complexity

## Conclusions

Biomarkers have the potential to transform the management of SCD in several ways. It is possible to screen patients early before they can be exposed to VOCs through the use of biomarkers, facilitating early intervention to prevent hospitalization. Using biomarkers could help identify severe VOC cases early on to guide toward more effective therapy and personalized medicine interventions. Using such biomarkers, researchers would detect the changes that would guide therapy adjustment and provide additional evidence on how well patients are responding to the therapies. Thereby, treatment responses can be monitored. In the process, biomarkers could also be useful in the identification of new therapeutic targets as well as measuring the efficacy of newly developed therapies. 
